# Former Training Relieves the Later Development of Behavioral Inflexibility in an Animal Model Overexpressing the Dopamine Transporter

**DOI:** 10.1007/s12035-022-03029-5

**Published:** 2022-09-20

**Authors:** Henriette Edemann-Callesen, Maximilian Glienke, Esther Olubukola Akinola, Maike Kristin Lieser, Bettina Habelt, Ravit Hadar, Nadine Bernhardt, Christine Winter

**Affiliations:** 1 Department of Psychiatry and Psychotherapy, Charité University Medicine Berlin, Campus Mitte, Charitéplatz 1, 10117 Berlin, Germany; 2 Department of Psychiatry and Psychotherapy, Medical Faculty Carl Gustav Carus, Technische Universität Dresden, Dresden, Germany; 3 Leibniz Institute of Polymer Research Dresden, Dresden, Germany

**Keywords:** Dopamine irregularities, Animal model, Cognitive deficits

## Abstract

A range of dopamine-dominating neuropsychiatric disorders present with cognitive deficits. In accordance, the dopamine transporter overexpressing rat model (DAT-tg rat) displays cognitive deficits by means of behavioral inflexibility and learning disabilities. It remains to be investigated when cognitive deficits emerge, due to the inherent DA irregularities, during the life course of the DAT-tg rat and what may relieve symptoms. The Morris water maze (MWM) was used to assess cognitive abilities in three cohorts of DAT-tg rats. In the first cohort, the development of cognitive deficits was assessed by repeatedly testing animals in the MWM at postnatal day (PND) 35, 60, and 90. In the second and third cohort, pharmacological interventions and transcranial direct current stimulation (tDCS) were tested in adult animals to understand what drives, and thus relieves, the deficits. Minor differences were observed between DAT-tg rats and control rats at PND 35 and 60, whereas cognitive deficits fully emerged at PND 90. A high dosage of methylphenidate diminished both behavioral inflexibility and improved learning abilities in adult rats. Interestingly, rats subjected early in life to the MWM also displayed improved behavioral flexibility as compared to rats naïve to the paradigm. Cognitive deficits gradually develop over time and fully emerge in adulthood. Pharmacological modulation of the ubiquitous DAT overexpression overall improves deficits in adult rats, whereas early training decreases later development of behavioral inflexibility. Thus, former training may constitute a preventive avenue that alters some aspects of cognitive deficits resulting from inherent DA abnormalities.

## Introduction

A stable and adequate dopamine (DA) signaling is required in the regulation of cognitive functions [1]. DA modulates and integrates the activity of several cortical and subcortical brain regions each of which is implicated in distinct aspects of cognitive processes including learning and memory [2, 3]. In the striatum, DA signals motivation and regulates the formation of associations, whereas in the hippocampus, DA drives selective attention and synaptic plasticity [4–7]. The information from subcortical areas is functionally integrated with, among others, the prefrontal cortex (PFC), which itself contains multiple DA projections and is involved in cognitive control [8]. Collectively, DA facilitates brain activity that enables a flexible adaptation of behavior based on memory and learned information, which is needed to tackle changing environments [9, 10]. The DA transporter (DAT) regulates DA homeostasis by controlling extracellular levels. Altered levels of DAT expression alongside DA irregularities are found in neuropsychiatric disorders in which also cognition is known to be affected, including attention-deficit hyperactive disorder (ADHD) and schizophrenia but also Tourette syndrome (TS) [11, 12]. The behavioral features observed in such neuropsychiatric disorders are considered to result from abnormal neural development [13–16]. The implications of DA irregularities on the development of cognitive deficits remain to be further explored. Here, the transgenic rat model that overexpresses the DAT (DAT-tg rat) allows for a direct investigation. The DAT-tg rat displays ubiquitous DAT overexpression in numerous areas, including the medial PFC (mPFC), striatum, hippocampus, thalamus, and nucleus accumbens. In adjacent to this, the DAT-tg rat displays specific up- and downregulation of dopamine receptors (DRD1 and DRD2) and altered DA content levels in areas specifically involved in cognition. Dopamine receptors are overexpressed in the hippocampus and striatum, yet downregulated in the mPFC. DA content levels are increased twofold in the hippocampus. On the contrary, levels are low in the striatum and paralleled with an increase in striatal monoamine oxidase (MAO) activity. In the striatum and hippocampus, distinct structural alterations are also found, including a decrease in the number of striatal interneurons and increased hippocampal volumes [17]. These profound imbalances in the DA system make the DAT-tg prone to cognitive deficits including learning and memory difficulties. Indeed, the DAT-tg rat displays cognitive deficits by means of learning deficits and behavioral inflexibility in the Morris water maze (MWM) [18]. DAT overexpression is further linked to repetitive behavior as also observed within a wide range of neuropsychiatric disorders. The DAT-tg rats display a repetitive grooming pattern which exacerbates following stress and amphetamine exposure. Collectively, the neurobiological alterations alongside the repetitive behavior and cognitive deficits found in the DAT-tg rat correlate with the clinical observations seen in TS [17, 18]. Here, we utilized the DAT-tg rat to investigate when, from juvenility to adulthood, the cognitive deficits appear, and which interventions may reverse these deficits. Specific drugs were applied to evaluate how manipulating distinct aspects of the DA system affected the deficits. To explore the repetitive nature of the behavior seen in the maze, the impact of anodal transcranial direct current stimulation (tDCS) and clonidine was assessed, as both have previously shown to reduce the repetitive behavior in the DAT-tg rat [17, 19].

## Materials and Methods

### Animals

We used male DAT-tg rats and their respective controls (wildtype (WT) rats) [17]. Rats were housed in a 12-h light/dark cycle (lights on at 6 a.m.), with food and water ad libitum. Animals that underwent surgery were single housed following the procedure. All efforts were made to reduce animal suffering and the numbers used. Experiments were conducted under the European Communities Council Directive and after approval by the local ethics committees.

### Morris Water Maze

A circular black pool (diameter 1.4 m) was filled with water to a depth of 33 cm. The water was made opaque by non-toxic white paint and the temperature was maintained at 24 °C (± 1 °C). Distal cues were provided around the pool. The pool was divided into four quadrants and a platform (14 × 14 cm) was placed in one quadrant of the maze. Animal behavior in the pool was videotaped and analyzed using EthoVision®XT by Noldus (Wageningen, Netherlands) and the Rtrack software package [20]. Testing consisted of an acquisition phase and a probe trial. During the acquisition phase, the platform was submerged below the water and animals could learn the location, through the use of distal cues and by releasing them from different starting points. The platform position quadrant had the following nomenclature: T (target), L (left from target), R (right from target), O (opposite target). The animal’s ability to locate the platform was tested in four trials per day with a 60-s trial limit over four consecutive days. After finding the platform, animals were allowed to remain for 5 s on the platform before being removed. Animals could rest for at least 15 min in between trials. On day 5, the platform was removed and one 60-s probe trial was performed. The success rate (% of trials) in finding the platform was computed for acquisition days. During probe trial, the time spent in the former target quadrant and platform crossings were recorded. Path length, swim velocity, and time animals spent close to the wall were assessed during all trials. Finally, the probability of using distinct search strategies to locate the platform was analyzed, including non-goal-oriented strategies (swimming close to the wall (i.e., thigmotaxis)), procedural strategies (i.e., scanning the pool), and contextual strategies (i.e., directly swimming towards the platform) for acquisition and probe trials. Maps were generated to visualize track densities and spatial search strategies.

### Pharmacological Treatment

Pharmacological interventions known to target different aspects of DA signaling were applied to assess the impact on performance in the MWM. The psychostimulant methylphenidate affects catecholamine transmission via regulation of DAT [21]. Lower dosages of methylphenidate (2.0 mg/kg) has shown to improve cognitive functions in male rats by distinctively activating the PFC, whereas higher dosages than this are believed to have more widespread effects throughout the brain [22]. Here, we chose to apply methylphenidate at both a lower and higher dosage than previously described to evaluate the differential effect between localized versus more widespread activation of brain areas in the DAT-tg rat. Methylphenidate was applied 15 min before MWM testing at a dosage of 0.5 (abbreviated MP 0.5) or 2.5 mg/kg subcutaneously (s.c) (MP 2.5), dissolved in 0.9% saline. To assess the impact of increasing DA content, levodopa (DO) was administered 30 min before MWM testing as a 20 mg/kg mixture in a ratio of 4:1 dissolved in 0.9% saline [23]. To evaluate the impact of DA receptors, the D2-receptor-antagonist haloperidol (HA) was applied 45 min before MWM testing, at a dosage of 0.30 mg/kg i.p, with a 0.2 M lactic acid vehicle [24]. To evaluate the repetitive nature of the behavior seen in the MWM, the alpha2-adrenoceptor-agonist clonidine (CL) was applied, as it has specifically been found to reduce repetitive behavior in the DAT-tg rat [17]. Clonidine was administered 20 min before MWM testing, at 0.01 mg/kg intraperitoneally (i.p), dissolved in 0.9% saline (SA) [17]. A group of WT- and DAT-tg animals were treated 15 min before MWM testing with 0.9% SA (i.p) and served as controls.

### Transcranial Direct Current Stimulation

#### Surgery

Animals were anesthetized using a cocktail of fentanyl (0.005 mg/kg), midazolam (2 mg/kg), and medetomidine hydrochloride (0.135 mg/kg). An epicranial electrode (2.1 mm diameter) was placed over the frontal cortex (AP + 2.0) [25] and fixed using autopolymerizing prosthetic repair material (Paladur; Kulzer GmbH, Hanau, Germany) and UV-induced light curing microparticle composite (Sinfony; 3 M Espe Dental AG, Seefeld, Germany). A small swab was placed in the epicranial electrode to prevent dirt from entering. For the counter electrode, the thorax was shaved to optimize the current application. Anesthesia was antagonized by a cocktail of naloxone (0.12 mg/kg), flumazenil (0.2 mg/kg), and atipamezole hydrochloride (0.75 mg/kg). Following surgery, animals were repeatedly observed for optimal recovery. Analgesia (meloxicam 0.2 mg/kg) was given for three consecutive days.

#### Stimulation

For delivery of tDCS, the epicranial electrode was filled with saline (0.9%) (contact area of 3.5 cm^2^) and a gold pin was inserted for stimulation application. A counter electrode (8 cm^2^; Physiomed Elektromedizin AG, Schnaittach, Germany) was placed on the thorax alongside electroencephalography (EEG) conductive paste (AC Cream, Spes Medica, Genova, Italy) and kept in place by a jacket (Lomir Biomedical Inc., Notre-Dame-de-l’Île-Perrot, Canada). Animals received 30 min of anodal (200 µA) or sham stimulation applied in the home cage starting 60 min before MWM testing. These settings have previously shown to reduce repetitive behavior in the DAT-tg rat by altering cortical activity levels and increasing striatal inhibitory properties [19]. We are thus confident that these settings are effective in the surpassing the thick scull of rats and alter neuronal activity. Stimulation was applied by a computer-interfaced current generator (STG4008 Multi Channel System GmbH, Reutlingen, Germany). The current strength was ramped for 10 s to prevent abrupt interruption and stimulation break effects. Animals receiving sham stimulation were connected to the system, yet no current was flowing.

#### Statistical Analysis

Data analysis was done using RStudio (version 3.6.1, Boston, USA) (RStudio Team (2020): Integrated Development for R. RStudio, PBC, Boston, MA URL http://www.rstudio.com/). The probability level of *p* < 0.05 was considered statistically significant for all tests.

##### Acquisition Phase

Performance measures success rate, path length, velocity, and time spent in the wall zone were analyzed by repeated measures ANOVA (aov, package: afex; group, MWM day*trial). Greenhouse–Geisser adjustment was used to correct violations of sphericity. For post hoc tests, we used Tukey’s method for multiple comparisons. Extraction of strategy usage was done using the Rtrack package and evaluated using binomial logistic regression (glmer, package: lme4) and odds ratio (OR).

##### Probe Trial

One-way ANOVA was used was used to test for statistical significance between experimental groups in path length, velocity, time spent in the wall zone, and platform crossings. In addition, time spent in different quadrants of the water maze was analyzed using two-sided *t*-test, in which the target quadrant was compared against the other quadrants. Test for equality of proportions without continuity correction was used for hippocampal strategy analysis in probe trials, with Holm adjustment for *p* values when applicable.

### Experimental Design

Experiments were conducted in three distinct cohorts of animals, in which (1) the development of cognitive deficits, (2) the effect of pharmacological interventions, and (3) the effect of transcranial direct current stimulation (tDCS) on MWM performance were assessed. To trace the emergence of cognitive deficits during the course of development, the same DAT-tg (*n* = 13) and WT (*n* = 12) animals were repeatedly testing at postnatal days (PND) 35, 60, and > 90. At each age, the MWM test was set-up with different platform positions to allow for repeated evaluation of learning the location of the hidden platform. The experiments performed at each age are thus considered to be independent experiments. The effect of pharmacological interventions and tDCS was tested in adult animals at PND > 90. Pharmacological interventions were applied to five groups of DAT-tg animals that received levodopa (*n* = 7), low dose methylphenidate (*n* = 7), high dose methylphenidate (*n* = 5), haloperidol (*n* = 8), or clonidine (*n* = 8), while a group of DAT-tg (*n* = 8) and a group of WT rats (*n* = 8) were administered saline and served as controls. Animals of the third cohort received either anodal tDCS (DAT-tg, *n* = 16; WT, *n* = 7) or sham stimulation (DAT-tg, *n* = 14; WT, *n* = 7).

## Results

### Performance in the MWM as Animals Mature

**Fig. 1:**
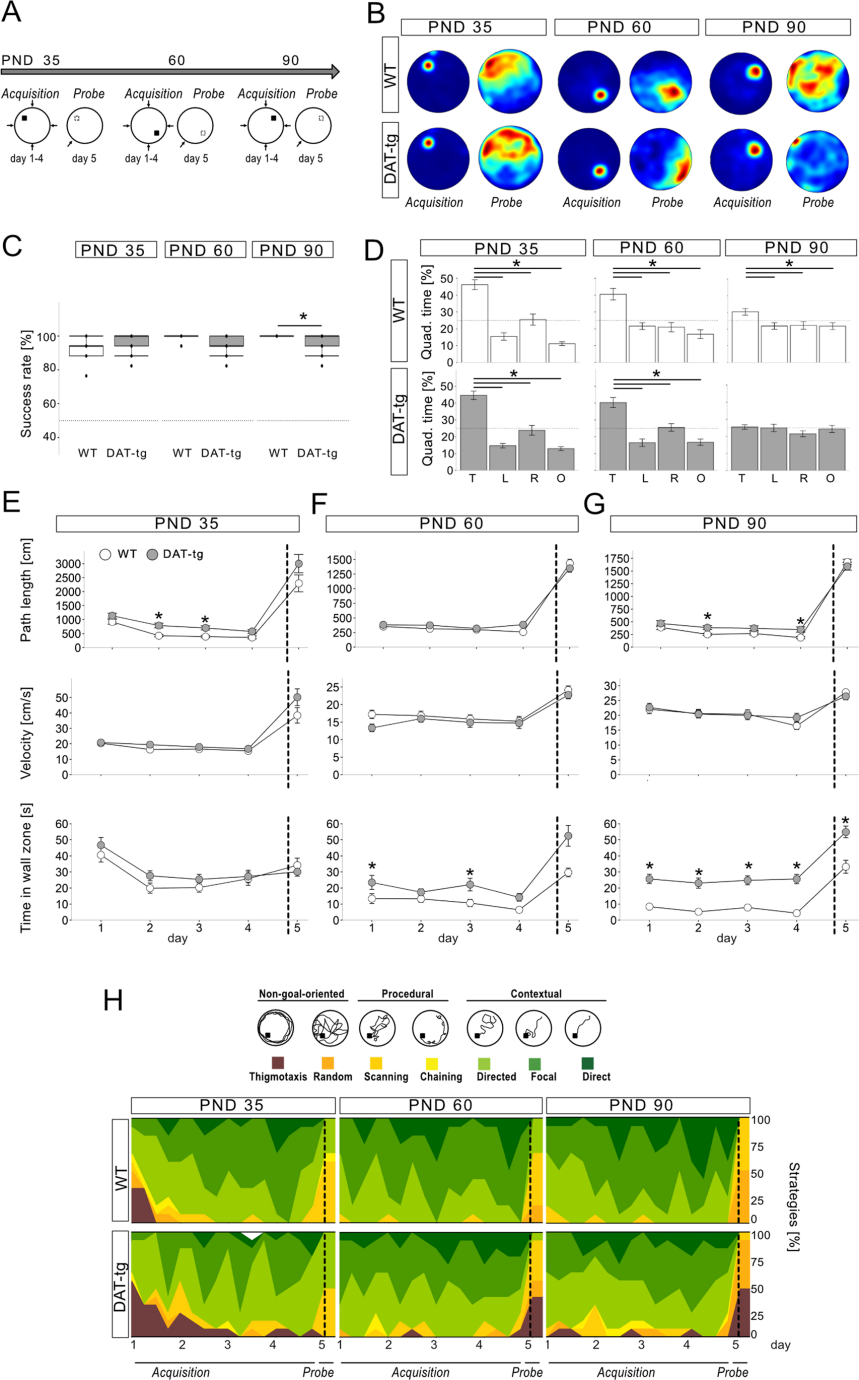
Former exposition to the Morris water maze enables DAT-tg rats to develop spatial search strategies. **A** Experimental layout to assess the effect of repeated MWM testing from postnatal day (PND) 35 to PND 90.** B** The group-wise probabilistic occupancy plots (red = high probability, blue = low probability) visualize intact acquisition (day 4) of platform position and spatial preference around the former platform position (day 5) for both genotypes.** C** Success rates in acquisition training prove the ability of DAT-tg rats to localize the hidden platform at adolescence and early adulthood. In full matured DAT-tg rats, success rate did not improve and was lower compared to WTs (*p* = 0.019).** D** Bar plots show percentage stay in each quadrant (T, target; L, left from target; R, right from target; O, opposite target) in probe trial and demonstrate quadrant preferences of both DAT-tg (gray) and WT (white) rats. While the latter clearly identified the former target quadrant at all three time points, adult transgenic rats did not display any preferences. Differences in time of residence were calculated with paired *t*-test against the target quadrant. **E**, **F**, **G** Path length (cm; top), velocity (cm/s, center), and time in wall zone (s, bottom) of DAT-tg and WT rats plotted separately for each study point. DAT-tg rats showed longer path length compared to WT rats on PND 35 (day 2: *p* = 0.003; day 3 *p* = 0.009) and PND 90 (day 2: *p* = 0.036; day 4: *p* = 0.01). WT rats were found to minimized their presence close to the wall as compared to DAT-tg during assessment at PND 60 (day 1: *p* = 0.048; day 3: *p* = 0.024) and PND 90 (all days: *p* ≤ 0.001). **H** Percentage distribution of used strategies in the MWM ranged from non-goal-oriented movement patterns like thigmotaxis and random searching to spatial and focused motion to the platform. Differences in strategy usage were calculated using a binomial regression model testing thigmotaxis versus other strategies. At PND 35 (*p* = 0.002, OR = 3.45), DAT-tg showed more thigmotaxis compared to WT over all testing days. Bars in **C**, **D**, **E**, **F**, and **G** represent SEM. *p < 0.05 assessed by repeated measure ANOVA (**C**, **E**, **F**, **G**). Dotted lines (**E**, **F**, **G**, **H**) separate acquisition from probe trial

During the acquisition phase, adolescent and early adult DAT-tg rats displayed similar rate of success in finding the hidden platform as compared to WT rats (PND 35 genotype: *F* = 1.83, *p* = 0.190; PND 60 genotype: *F* = 3.19, *p* = 0.087), whereas the rate of success was significantly reduced once DAT-tg rats reached adulthood (PND 90 genotype: *F* = 6.39, *p* = 0.019) (Fig. 1A–C). During the probe trial both adolescent and early adult DAT-tg rats spent the majority of time in the former target quadrant (PND 35 paired *t*-test all *p* ≤ 0.001; PND 60 paired *t*-test all *p* ≤ 0.0001), whereas no preference was detected in the adult DAT-tg rats (PND 90 WT paired *t*-test all *p* ≤ 0.05: DAT paired *t*-test all *p* > 0.05) (Fig. 1D). Adolescent and adult DAT-tg rats displayed an increased preference for the wall of the maze (PND 60 genotype: *F* = 11.66, day *F* = 18.93, *p* < 0.001; PND 90 genotype: *F* = 33.07, *p* < 0.001, day *F* = 44.40, *p* < 0.001). Adolescent and adult DAT-tg rats displayed a longer path length compared to WT rats (PND 35: genotype: *F* = 10.09, *p* = 0.004; day: *F* = 28.05, *p* < 0.001, trial: *F* = 7.62, *p* < 0.001; PND 90 genotype: *F* = 8.53, *p* = 0.008; day *F* = 6.07, *p* = 0.002). There was no difference in swim velocity between groups at any of the time points tested (PND 35 genotype: *F* = 0.61, *p* = 0.441; PND 60 genotype: *F* = 0.92, *p* = 0.348; PND 90 genotype: *F* = 0.19, *p* = 0.670) (Fig. 1E–G).

**Fig. 2:**
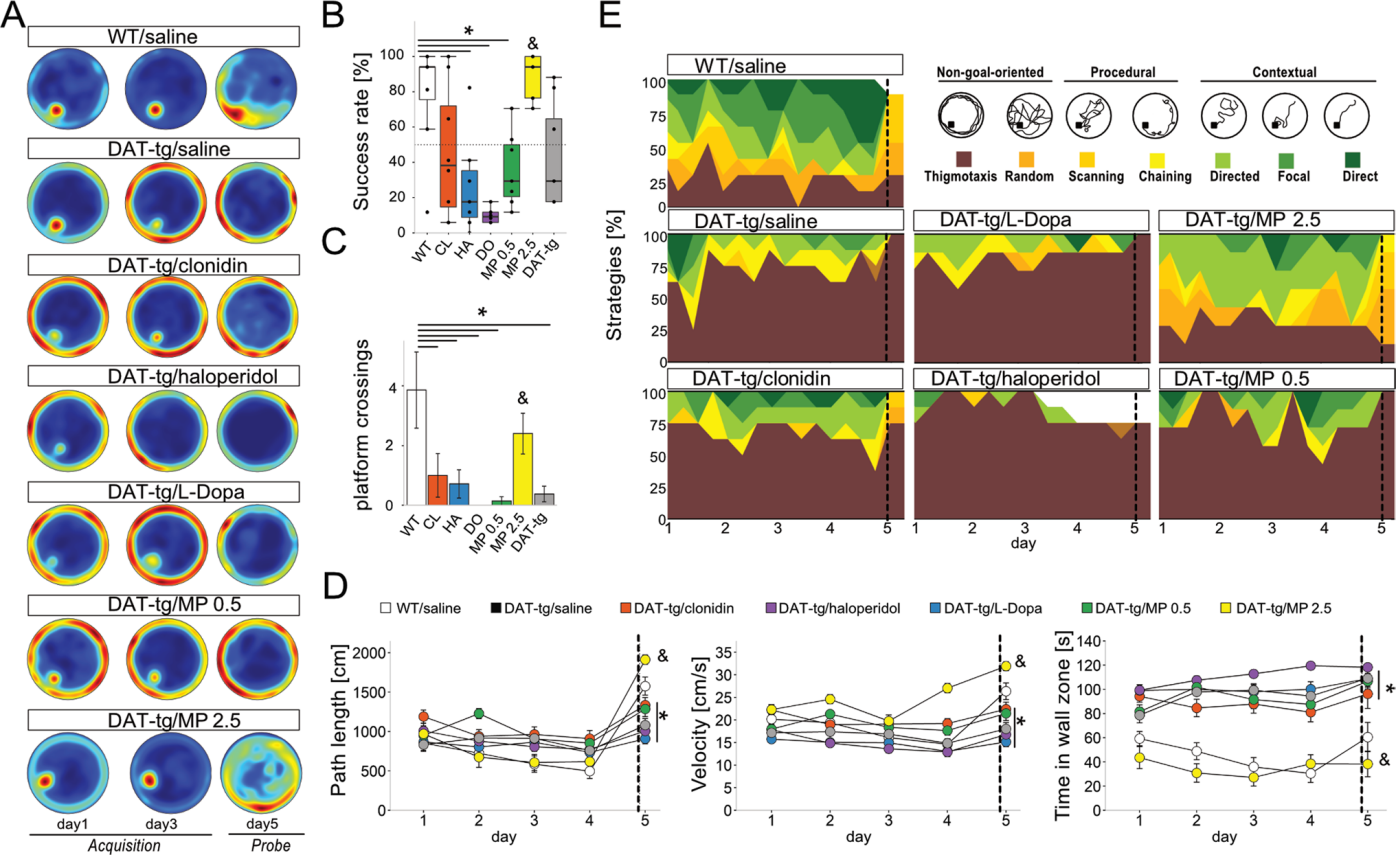
High dose methylphenidate improves MWM performance in adult DAT-tg rats.** A** Group-wise probabilistic occupancy plots (red = high probability, blue = low probability) demonstrate the development of place-specific preferences for the platform position during MWM assessment in WT/saline and DAT-tg/MP 2.5 (2.5 mg “high” dose methylphenidate) rats, while all other groups fail to acquire sufficient knowledge of platform position. **B** WT and DAT-tg/MP 2.5 rats show a comparable high success rate during acquisition training (WT vs. MP 2.5: *p* = 0.991) and **C** number of platform crossings in the probe trial (WT vs. MP 2.5: *p* = 0.776). **D** Path length (cm; left), velocity (cm/s; middle), and time in zone wall (s; right) further illustrate the treatment effect and similarities in regard to wall zone time between WT and DAT-tg/MP 2.5 rats, while DAT-tg rats in all other group present spent most of their time swimming close to the wall (WT vs. MP 2.5: *p* = 0.991; DAT-tg vs. MP 2.5: *p* = 0.002). **E** Movement patterns in the water maze were classified from non-goal-oriented like thigmotaxis to contextual search strategies like focal or direct search towards the target platform. As previously shown [18], adult WT but not DAT-tg rats employ advanced spatial search strategies indicating a hippocampal involvement. When thigmotaxis was tested against all other strategies in a binomial logistic regression model, only DAT-tg/MP 2.5 rats showed a similar strategy use compared to WT (MP 2.5 vs. WT: *p* = 0.349; MP 2.5 vs. DAT-tg *p* < 0.001). *p < 0.05 assessed by repeated measure ANOVA (**D**) respectively ANOVA (**B**, **C**). Error bars in **B**, **C**, **D** show SEM. Dotted lines (**D**, **E**) separate acquisition from probe trial

**Fig. 3:**
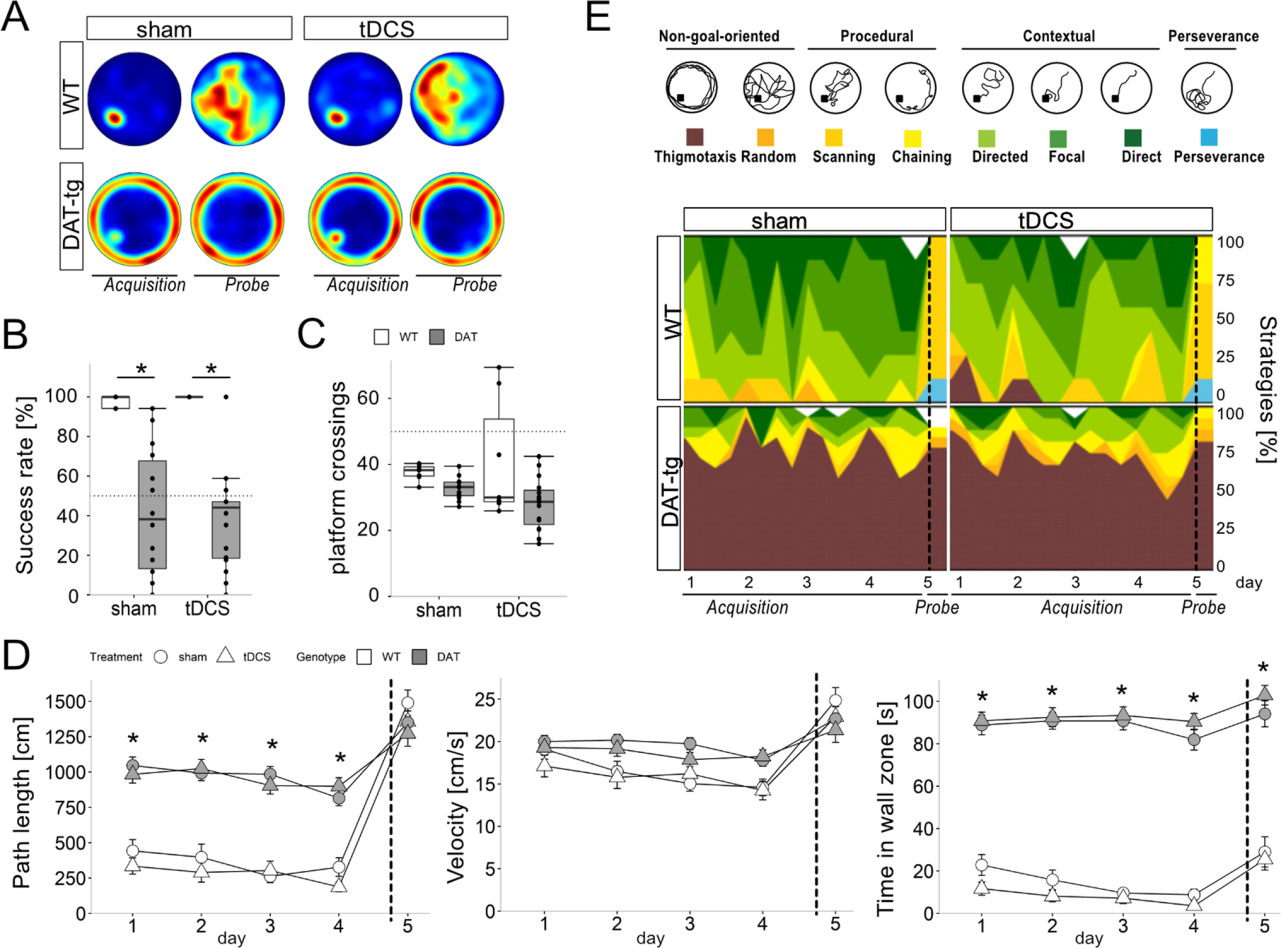
tDCS does not facilitate spatial learning in adult DAT-tg rats. **A** Group-wise probabilistic occupancy plots (red = high probability, blue = low probability) visualize the preferences for the platform position during acquisition training (day 4) and former platform position during probe trial (day 5) in WT animals. DAT-tg rats however swam mostly in more peripheral zones. **B** Box-Whisker plots emphasize the difference regarding success rates between WT and DAT-tg rats independent of their treatment (*p* < 0.001).** C** Platform crossings in probe trial underline the lack of spatial memory in DAT-tg rats. WTs remembered the locality of the platform and thus crossed the former position more often (*p* < 0.001). tDCS treatment did not enable DAT-tg rats to improve their spatial memory.** D** Path length (left, cm), velocity (center, cm/s), and time in wall zone (right, s) indicate shorter swimming distance in acquisition training (*p* < 0.001) and less time spent in the area close to the wall (*p* < 0.001) for WT rats whereas swimming speed displayed no differences between groups. **E** Color-coded plots represent strategy usage under acquisition training and probe trials. Thigmotaxis as rather instinct-driven movement (brown) was tested against spatial search strategies in a binomial logistic regression model. Note the lack of thigmotaxis in WTs compared to DAT-tg rats (*p* < 0.001) independent of the treatment. Bars in **B**, **C**, and **D** represent SEM. *p < 0.05 assessed by repeated measure ANOVA (**B** and **D**) and ANOVA for probe trial data on day 5 (**B**, **C**, and **D**). Dotted lines (**D**, **E**) separate acquisition from probe trial

When search strategies were assessed, adolescent DAT-tg rats displayed a higher chance of choosing thigmotaxic behavior as compared to WT rats (PND 35 genotype *z* = 2.97, OR = 3.45, *p* = 0.003). When behavior was plotted over the course of days, thigmotaxic behavior was especially present in both WT rats and DAT rats during the first days of testing. Interestingly, once animals reached adolescence and adulthood, the use of thigmotaxic behavior was low and DAT-tg rats employed spatial search strategies comparable to the WT rats (PND 60 *z* = 0.005, *p* = 0.996; PND 90 *z* = 0.008, *p* = 0.993) (Fig. 1H). These results are surprising when compared to both, previous findings [18] and to the adult DAT-tg rats in the present study serving as controls (sham and saline groups) in which thigmotaxic behavior in both cases was the dominating strategy (Fig. 2E, Fig. 3E). This indicates that exposing animals to the MWM from an early age may enable them to develop more successful search strategies as compared to adult DAT-tg rats who are naïve to the paradigm.

### The Effect of Pharmacological Interventions in the Adult DAT-tg Animal

WT rats and DAT-tg rats who had been exposed to MP displayed a comparable rate of success in finding the hidden platform (genotype: *F* = 6.66, *p* < 0.0001, post hoc *p* = 0.991) (Fig. 2A–B) and platform crossings (genotype: *F* = 4.50, *p* = 0.001, post hoc *p* = 0.776) (Fig. 2C). The WT rats and DAT-tg rats exposed to MP further displayed similar path lengths (genotype: *F* = 11.99, *p* < 0.001, post hoc *p* = 0.338), swim velocity (genotype: *F* = 12.39, *p* < 0.001, post hoc *p* = 0.332), and time spent close to the wall (genotype: *F* = 7.92, *p* < 0.001, day *F* = 6.55, *p* < 0.001, post hoc *p* = 0.991). DAT-tg rats in all other groups spent the majority of time close to the wall (Fig. 2D). MP increased the chance by 13 times of employing more elaborate strategies as opposed to thigmotaxis (comparison to DAT-tg saline: *z* = 7.143, OR = 12.94, *p* = 9.12e − 13), whereas application of HA further increased the use of thigmotaxis (comparison to DAT-tg saline: *z* =  − 4.109, OR = 0.19, *p* = 3.97e − 0.5) (Fig. 2E). Exposure to the remaining pharmacological interventions did not have any impact on search strategies in the MWM.

### The Effect of tDCS in the Adult DAT-tg Animals

WT rats displayed a higher success in finding the platform (genotype: *F* = 59.09, *p* ≤ 0.0001), spent more time in the target quadrant (genotype *F* = 10.25, *p* = 0.00271), and crossed the goal zone more frequently as compared to DAT-tg rats, regardless of the type of stimulation applied (genotype *F* = 59.41, *p* ≤ 0.0001) (Fig. 3A–C). DAT-tg rats displayed a longer path length (genotype: *F* = 63.24, *p* ≤ 0.001, day: *F* = 5.34, *p* = 0.003; trial *F* = 33.84, *p* ≤ 0.001) and spent more time close to the wall (genotype: *F* = 167.34, *p* ≤ 0.001, day: *F* = 11.59, *p* < 0.001; genotype:day *F* = 9.84, *p* < 0.001). There was no difference in swim velocity between groups (Fig. 3D). DAT-tg rats displayed high levels of thigmotaxic behavior irrespective of the type of stimulation applied (acquisition trial: *z* =  − 6.34, OR = 0.009, *p* < 0.001), whereas the WT rats did not display this behavior (probe trial: *X*-squared = 24.548, *p* ≤ 0.0001, tDCS WT 0%; sham WT 0%; tDCS DAT-tg 86.6%; sham DAT-tg 71.4%) (Fig. 3E).

## Discussion

We here find that the cognitive deficits observed in DAT-tg rats gradually emerge and become fully evident once animals reach adulthood. Of the interventions investigated, solely a high dose of methylphenidate overall improved cognitive deficits in the adult rats. Interestingly, the animals who had repeatedly been subjected to training from an early age displayed less inflexible behavior. This indicates that for some aspects of the cognitive deficits observed in the DAT-tg rat, former training has the potential as a preventive avenue.

### Former Training Decreases Behavioral Inflexibility in the DAT-tg Rats

The cognitive deficits observed by the adult DAT-tg rats in the MWM are twofold as they include (1) spatial learning difficulties as adult animals are unable to successfully learn the location of the hidden platform and (2) behavioral inflexibility as adult animals display profound thigmotaxic behavior indicating an inability to perform goal-directed behavior [18]. In the present study, the same batch of DAT-tg rats were repeatedly tested in the MWM as they matured, to evaluate when the cognitive deficits emerge. At adolescence (PND 35) and early adulthood (PND 60), the DAT-tg rats exhibited the same rate of success in finding the hidden platform and the same level of reference memory, as measured by the time spent in the former target quadrant as control animals. This was despite the fact that adolescent DAT-tg rats (PND 35) displayed an increase in thigmotaxic behavior and path length as compared to controls. Once reaching full maturity (PND > 90), a decline in performance was found, as DAT-tg rats now showed lower success in finding the platform and a reduction in reference memory. As such, we here find that the DAT-tg rats’ ability to successfully solve the MWM gradually decreases as animals mature. Of note, other behavioral features such as repetitive behavior in the DAT-tg rats are also found to develop over time, as it becomes visible from PND 60 and continues to intensify towards PND 95 (Bernhardt et al., in preparation). Other animal studies have shown that normal cognitive functioning in adulthood relies on a maturation of the mesocortical DA system that takes place during late adolescence [26, 27]. The overexpression of DAT is present in the DAT-tg rats from birth, yet given that the cognitive deficits only fully emerge in adulthood, it remains to be investigated which pathophysiological processes take place as the rats mature and whether adolescence here also constitutes a critical time period in the transition towards cognitive deficits.

Most interestingly, however, our results show that adult DAT-tg rats who had been subjected to former training in the MWM were now able to choose more flexible behavioral search strategies once reaching adulthood. These results are in contrast to our previous findings in which immense thigmotaxic behavior was the dominating strategy in the adult DAT-tg rats during all days of testing [18]. Thigmotaxic behavior is a common, initial behavior observed in rats, yet as animals adapt to the maze this behavior is normally replaced by other more efficient search strategies. In accordance, we also find that WT rats initially display thigmotaxic behavior during the first trials, yet this is swiftly replaced by the use of spatial search strategies. Performance in the MWM relies not only on the ability to learn the spatial location of the platform, but also requires that animals are flexible in their behavioral response and are able to plan and execute the behavior needed to successfully get to the platform [28]. We here show that exposing DAT-tg rats to early training has the potential of diminishing behavioral inflexibility later in life, thus improving some of the required abilities needed to succeed in the MWM. However, despite this improvement, early training did not translate into animals successfully identifying the platform, as DAT-tg rats continued to display low success rate and deficits in reference memory once reaching adulthood. As such, early training may enable the animals to adapt their behavior to their surroundings, yet this improvement is not sufficient to ultimately solve the maze, thus indicating persistent learning deficits. Adequate performance in the MWM is found to rely on a coordinated activity between several brain areas, including the striatum, hippocampus, and frontal cortex, in which the DA system has a regulatory role [28, 29]. As the DAT-tg rats display DA irregularities within all the above-mentioned brain areas, it is not surprising that adult animals fail to ultimately solve the task in the MWM. The dissociation we report following early training in the DAT-tg rat, in which behavioral flexibility improves yet learning deficits persists, shows that early training may influence some but not all of the brain areas needed to successfully perform in the MWM. The mPFC and dorsomedial striatum are found to form a corticostriatal circuit involved in goal-directed behavior and thus may be involved in selecting the appropriate behavioral strategy needed in the MWM [28, 30–32]. In accordance, when tested in the MWM, rats with lesion to the PFC are incapable of planning and performing the required course of movements needed to get to the platform [33] and inflexible behavior by means of thigmotaxic behavior is induced in rats following specific lesion to the dorsomedial striatum [34]. On the other hand, the hippocampus has long been considered to play a central role in the spatial learning aspects of the MWM [28]. As such, the selective improvement in behavioral flexibility following early training in the DAT-tg rats may result from a positive effect on DA signaling within corticostriatal circuits, while at the same time reflecting an inability to modulate other areas such as the hippocampus by which spatial learning deficits persist.

### Methylphenidate at a Higher Dosage Relieves Cognitive Deficits in Adulthood

Given the regulatory role of DA in MWM performance and cognitive functioning in general, we chose to apply DA modulating drugs prior to adult animals having to perform in the MWM, to determine what aspects of DA signaling drive the cognitive deficits seen in adult rats. Methylphenidate is known to increase catecholamine neurotransmission by interference with DAT [21]. Here, we applied methylphenidate at a low and high dose to investigate how modulating DAT activity, either locally or more broadly throughout the brain, influences behavior in the MWM. In the DAT-tg rat, DAT overactivity causes rapid clearing of DA from synapses leading to synaptic DA deficiency as well as changes in DA receptor availability [17]. We applied levodopa and haloperidol to access how interfering with the consequence of DAT overexpression impacts performance in the MWM. Levodopa is an amino acid precursor of DA that increases synaptic levels of DA [35]. Haloperidol is an antipsychotic that works as a D2-receptor antagonist [36]. Solely the application of methylphenidate led to improved performance. Dosage was of importance, as a higher dosage was needed (2.5 mg/kg) whereas a lower dosage (0.5 mg/kg) had no effect. Methylphenidate has been shown to improve spatial working memory in healthy subjects as well as in children and adults with ADHD [21, 37–39]. Methylphenidate is known to block the reuptake of DA by acting on DAT, thus increasing the synaptic DA availability in areas such as the hippocampus, striatum, and PFC. This effect has been coupled with an improvement in the attention needed to successfully perform spatial learning and memory processes [21, 22, 40, 41]. As such, the overall improvement in performance following the application of a higher dose of methylphenidate in the DAT-tg rat may reflect the need for interfering with DAT activity in several implicated brain areas, in order to reverse all aspects of the cognitive deficits seen in the MWM. It is shown in rats that DAT mainly plays a central role in regulating extracellular DA in the striatum, whereas the norepinephrine transporter (NET) plays a large role in regulating DA levels in the mPFC [42]. As methylphenidate acts on both DA and norepinephrine uptake, future studies investigating the effect of selective DAT and NE inhibitors in the DAT-tg rat are necessary for further increasing our understanding on how methylphenidate mediates its positive effect on the cognitive deficits. Application of the D2-receptor antagonist haloperidol further exacerbated the occurrence of thigmotaxic behavior in the DAT-tg rats. Such increase in thigmotaxic behavior following blockage of D2 receptors by haloperidol is suggested to reflect an induced inability to choose the strategy needed to solve the task [43]. Others have found that blocking D2 receptors specifically in the prefrontal cortex leads to inflexible behavior [44]. Mice who selectively overexpress D2 receptors in the striatum display persistent changes in PFC functioning, resulting in behavioral inflexibility [45]. As DAT-tg rats display low levels of D2 receptor expression in the mPFC, yet increased levels in the striatum, D2 receptors may indeed play a role in the observed thigmotaxic behavior. The influence of D1 receptors on behavior in the DAT-tg rats still needs to be assess; however, it is not unlikely that a combined effect of D1 and D2 receptors are involved in the cognitive deficits, as D1 and D2 receptors are considered to work in concert when it comes to behavioral flexibility and cognitive control [30]. Collectively this suggests that the altered expression levels of DA receptors, as a consequence of DAT overexpression, may work as a driving force especially within the corticostriatal circuit to produce the thigmotaxic behavior seen in the DAT-tg rat. In contrast to these findings, levodopa had no impact on performance in the MWM, indicating that a direct increase in DA levels is not sufficient to counterbalance the widespread deficits induced by DAT overexpression.

Apart from an inadequate ability to select an appropriate strategy, it has also been proposed that thigmotaxic behavior in the DAT-tg rats rather reflects stress-induced repetitive behavior, as the inherent DA irregularities increase the animals’ susceptibility towards such behavior [18]. The application of clonidine and anodal tDCS applied to the frontal cortex have previously been shown to successfully reduce repetitive behavior in the DAT-tg rat [17, 19]. Therefore, both interventions were applied in the present study, to further disentangle the repetitive nature of the behavior seen in the MWM and to test the therapeutic potential. Here, we show no effect of clonidine or tDCS on any of the features investigated in the MWM, as DAT-tg rats continued to display poor spatial learning and heightened thigmotaxic behavior. Either this suggests that the observed thigmotaxic behavior does not reflect repetitive behavior or that the thigmotaxic behavior represents a different mode of repetitive symptoms that differ from those previously observed. Nevertheless, whether the thigmotaxic behavior is a consequence of an inadequate ability to choose an efficient strategy or rather due to a restriction and rigidity in behavior as defined by repetitive symptoms, it overall reflects behavioral inflexibility. As mentioned, both clinical and preclinical data demonstrate that the ability to adapt to changing environments relies on functional changes in DA signaling [29, 46–48]. Neither anodal tDCS that has previously been tested in the DAT-tg rats nor clonidine is primarily effective via the DA system, which may account for the lack of effect of these interventions in MWM [19]. Other studies have shown that the application of the atypical antipsychotic drug risperidone as well as tDCS before symptom manifestation prevents later symptoms in an animal model of schizophrenia [49, 50]. As symptoms only start to emerge in adulthood, it remains to be investigated whether the interventions tested in the present study would have a stronger impact in the DAT-tg rat, if applied before the manifestation of symptoms.

As it currently stands, the neurobiological alterations and behavioral profile of the DAT-tg rat are found to correlate with clinical observations primarily seen in TS [51]. However, the DAT-tg offers more than a model of one distinct disorder, as it constitutes an experimental framework in which the pathological implications of DAT overexpression and its link to a range of neuropsychiatric symptoms in general can be investigated. As seen in the present study and consistent with the neurodevelopmental nature of some neuropsychiatric disorders, we find that DAT overexpression lays the foundation for negative behavioral trajectory, in which abnormal behavior slowly, yet gradually, arises as the animals mature. To sufficiently reverse the different aspects of cognitive deficits, we find that DAT activity potentially has to be modulated in several brain regions. However, we also find that early training selectively prevents behavioral inflexibility as animals mature. As such, former training may constitute a preventive approach for neuropsychiatric disorders presenting with behavioral inflexibility. This is indeed the case for TS in which symptoms often develop over time, potentially due to an imbalance in the regulation between goal-directed behavior and habit formation [52, 53]. For these patients, who are often young when symptoms emerge, the application of an early, non-pharmacological treatment approach that decreases the later burden of symptoms may be appealing.

## Conclusion

In the DAT-tg rat, altered DAT expression levels and subsequent DA irregularities lead to the inability to solve the task at hand. These deficits are not fully present in adolescent animals, indicating that symptoms gradually develop as animals mature. A high dosage of methylphenidate known to modulate the DAT in several brain areas diminishes both behavioral inflexibility and learning deficits in adult rats, whereas training of adolescent animals decreases behavioral inflexibility later in adulthood. As such, former training may constitute a non-pharmacological, preventive avenue that has the potential of altering some aspects of cognitive deficits seen in neuropsychiatric disorders due to inherent DA abnormalities.

## Data Availability

The datasets generated during and/or analyzed during the current study are available from the corresponding author on reasonable request.
